# How social media usage affects psychological and subjective well-being: testing a moderated mediation model

**DOI:** 10.1186/s40359-023-01311-2

**Published:** 2023-09-22

**Authors:** Chang’an Zhang, Lingjie Tang, Zhifang Liu

**Affiliations:** 1 School of Foreign Studies, Xi’an Jiaotong University, No. 28 Xianning West Road, Beilin District, Xi’an, 710049 Shaanxi Province China; 2 Educational Studies, Universiti Sains Malaysia, Bangunan D02, 11800 Gelugor City, Penang Island Malaysia

**Keywords:** Social media, Cyberbullying, Self-esteem, Online social support, Psychological well-being, Subjective well-being, Moderated mediation

## Abstract

**Background:**

A growing body of literature demonstrates that social media usage has witnessed a rapid increase in higher education and is almost ubiquitous among young people. The underlying mechanisms as to how social media usage by university students affects their well-being are unclear. Moreover, current research has produced conflicting evidence concerning the potential effects of social media on individuals' overall well-being with some reporting negative outcomes while others revealing beneficial results.

**Methods:**

To address the research gap, the present research made an attempt to investigate the crucial role of social media in affecting students’ psychological (PWB) and subjective well-being (SWB) by testing the mediating role of self-esteem and online social support and the moderation effect of cyberbullying. The data in the study were obtained from a sample of 1,004 college students (483 females and 521 males, *M*_age_ = 23.78, *SD* = 4.06) enrolled at 135 Chinese universities. AMOS 26.0 and SPSS 26.0 as well as the Process macro were utilized for analyzing data and testing the moderated mediation model.

**Results:**

Findings revealed that social media usage by university students was positively associated with their PWB and SWB through self-esteem and online social support, and cyberbullying played a moderating role in the first phase of the mediation process such that the indirect associations were weak with cyberbullying reaching high levels.

**Conclusion:**

These findings highlight the importance of discerning the mechanisms moderating the mediated paths linking social media usage by young adults to their PWB and SWB. The results also underline the importance of implementing measures and interventions to alleviate the detrimental impacts of cyberbullying on young adults’ PWB and SWB.

## Introduction

In this digital world, the utilization of social media has become a massive and meaningful part of our everyday life and has grown substantially in recent years [1, 2]. People of all ages, adults and adolescents, utilize a diverse array of social media platforms to engage in meaningful connections, both in intimate settings with loved ones and in expansive networks encompassing friends, acquaintances, and professional peers [3]. It is worth emphasizing that the younger generation is dedicating an ever-growing portion of their time to engaging in online networking platforms, indulging in e-games, exchanging messages, and immersing themselves in various forms of social media [4]. As a result, there is growing attention among the scholars of social sciences paid to social media research. Despite a handful of studies that have been conducted to shed light on the reasons behind the excessive usage of social media, still literature exploring the potential consequences of utilizing social media is limited, particularly among college students in the context of China. Taking up this research gap, we intend to examine the effects of social media usage on students’ wellbeing, for example, PWB and SWB, which are two distinct but related dimensions of well-being.

Studies on well-being have been grounded on two different philosophical approaches: the hedonic perspective, which defines well-being as the pursuit of pleasure and avoidance of pain, and the eudaimonic perspective, which conceptualizes well-being as the extent to which an individual achieves their potential and experiences personal growth [5]. Most studies on the hedonic psychological perspective have focused on using SWB measures [6], whereas the eudaimonic approach, as proposed by Ryff [7], includes a multidimensional model of PWB consisting of six different aspects of positive functioning: autonomy, environmental mastery, personal growth, positive relations with others, purpose in life, and self-acceptance [8]. Although researchers have different approaches, they generally agree that well-being should be understood as a complex concept that incorporates elements from both the hedonic and eudaimonic perspectives [5, 9]. Moreover, many scholars recommended that both concepts of wellbeing be re-examined by conducting in-depth and larger research subjects involving diverse cultures and countries [10]. This is necessary and meaningful since existing studies are typically conducted with subjects in countries referred to as WEIRD (Western, Educated, Industrialized, Rich, Democratic). As such, in this study, we attempted to investigate the impact of social media usage on both PWB and SWB.

Existing literature has revealed that the use of social media is closely related to individuals’ well-being. Some studies found that social media usage can produce beneficial effects. For instance, social media can increase users’ sense of connectedness with others [4], thus reducing social isolation. Some other studies have demonstrated that engaging in social interactions through smartphones exquisitely enhances one's overall sense of well-being, as it remarkably diminishes feelings of loneliness and shyness [11] while providing a sense of intimacy [12], and mobile voice communication with loved ones is a powerful predictor of enhanced PWB [13]. Furthermore, numerous studies have revealed that the utilization of entertainment-motivated social media can help improve users’ self-disclosure [14], and facilitated social connections through social media platforms can decrease the sense of stigmatization [15] and enhance belongingness and social inclusion [16], contributing to increased SWB. However, some researchers have stressed that social media usage can occasionally divert users' attention from meaningful relationships and hinder social interactions [17, 18] and a number of scholars have cautioned against the potential additive relationship with digital devices like smartphones if used excessively [12, 19], possibly due to the fear of missing out [20]. The utilization of social media has unfortunately been linked to a range of distressing consequences including heightened feelings of anxiety [21], profound loneliness [22], and debilitating depression [23]. Additionally, it has been found to perpetuate a sense of social isolation, as well as engender a phenomenon known as "phubbing," whereby individuals become excessively engrossed in their smartphones, thereby compromising genuine interpersonal connections during in-person interactions [24].

The inconsistent research findings regarding the impact of social media on individuals’ well-being suggest that some factors may play a role in this mechanism. Actually, in addition to the direct association between social media usage and well-being, a number of studies have further identified mediators to investigate underlying mechanisms of this relationship. Previous studies have identified self-esteem and online social support as two promising mediators of the link between social media usage and PWB and SWB. And empirical studies have revealed that media attention and dependency were proven to improve individuals’ self-efficacy [25], thus increasing their self-esteem. Most importantly, people would rely more on social media, especially during the COVID-19 pandemic in China [26], to seek social support via the Internet as in-person social support was seriously reduced [27]. Moreover, social media usage like for informational uses was found to increase people’s self-esteem [28] and can provide an important avenue for obtaining online social support from friends, peers and important others [29], which, in turn, reinforce peoples’ PWB and SWB. Although previous studies on mediation effects of self-esteem and online social support have helped elucidate the complex relationship between social media and well-being, further exploration can be made. To test the concurrent mediating effects of self-esteem and online social support, which have been investigated separately in prior studies, would shed more light on the interplay between social media usage and well-being. Furthermore, researchers have acknowledged the importance of exploring the generalizability of their findings to different cultures, like Asian cultures, particularly Chinese culture where collectivism runs strong [30]. Because previous research indicated that individuals who recorded high collectivism were apt to experience higher levels of well-being, regardless of social media usage [15], suggesting that a hierarchical society with a strong collectivist culture can play an important role in the impact of people’s social media use on their well-being.

Another factor that intrigued us is cyberbullying. A review of literature on this topic concluded that cyberbully is prevalent on the Internet and some 11.2% to 56.9% of Chinese adolescents reported experiences of cyberbullying victimization, the second-highest median rate among nine nations surveyed in the study [31]. Similar to traditional bullying, cyberbullying as a victim via social media is founded to be closely related to a series of behavioral and psychological problems (e.g., depression, anxiety, post-traumatic stress disorder, and suicidal ideation) [32, 33]. Cyberbullying victimization has also been found to reduce individuals’ self-esteem [34] and make them feel less inclined to engage with social media platforms and online communities [35], thus decreasing online social support from peers, friends, and family members. This analysis inspired us to examine whether cyberbullying acts as a moderator in the association between social media usage and well-being. Given the widespread occurrence and undesirable effects of cyberbullying, it is significant for scholars to explore its underlying mechanisms and underexamined consequences. Meanwhile, previous empirical investigations on cyberbullying have largely focused on children and teens [36]. There have been comparably fewer studies on the influence of cyberbullying on mental health among young adults, like college students, especially in China. In addition, cyberbullying may have a differential impact on adults vs.children. This is particularly true for cyberbullying on social media, as there are differences in the amount of time spent on social media and the specific platforms used by children and adults [37].

**Fig. 1:**
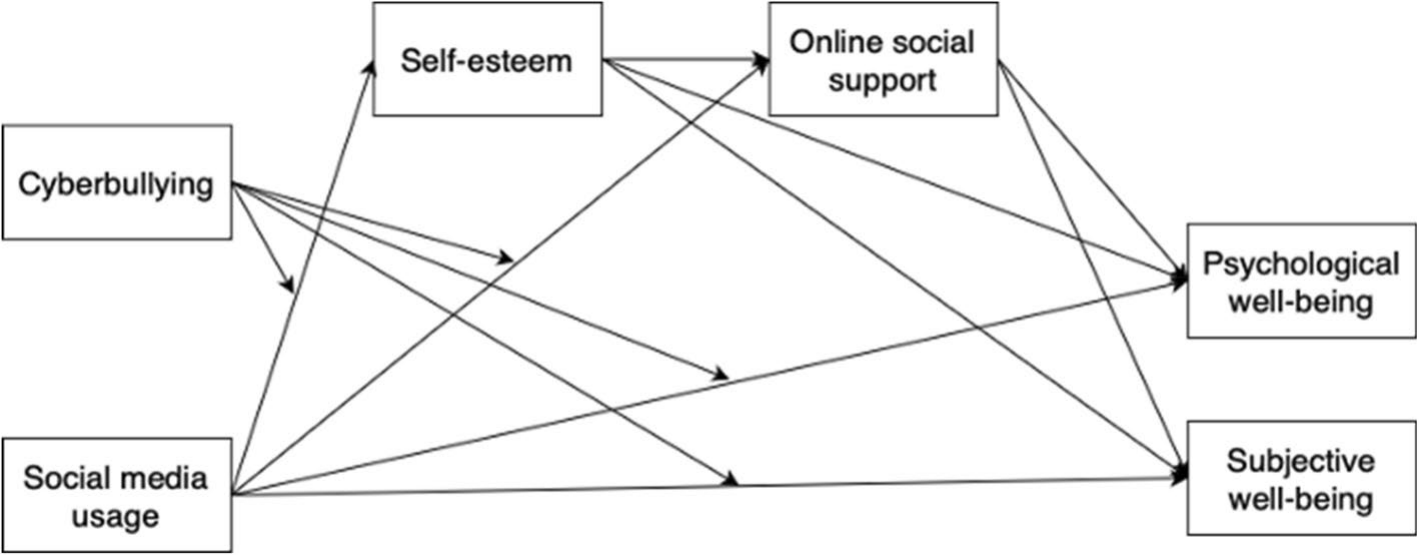
Proposed moderated mediation model

Against the above background and in line with previous studies [16, 38–40] we formulated a moderated mediation model to test the roles of self-esteem and online social support as mediators and cyberbullying as a moderator in the relationship of social media and PWB and SWB. Figure 1 presents our moderated mediation model.

## Literature review and hypotheses development

### Students’ social media usage and well-being

University students utilize the Internet for various reasons, including leisure activities like participating in online communities or playing games, educational tasks such as completing assignments or applying for scholarships, and practical activities such as researching companies for job interviews. Previous studies have unveiled the rising popularity of social media among students, while more recent investigations have underscored the profound impact that the usage of social media has on their PWB and SWB [41, 42]. Research studies have observed a directly or indirectly positive relationship of social media usage with students’ PWB [43, 44] and SWB [41, 42]. Specifically, PWB serves as a crucial determinant of the overall quality of life, referring to individuals' emotional states and appraisals of their existence [45], and can include a multiple of dimensions such as autonomy, environmental mastery, personal growth, positive relations with others, purpose in life, and self-acceptance [8]. The utilization of social media by students offers them a broader platform to voice their opinions and emotions regarding their rights, fostering their self-assurance and confidence, and bolstering their knowledge and understanding [46]. During times of crisis like during the period of COVID-19, the utilization of social media platforms by students presents a valuable avenue for stress relief as they can openly express their thoughts and receive advice from others on how to navigate and overcome the challenging circumstances they find themselves in [47]. In addition, researchers have also revealed that students’ frequent social media usage to exchange thoughts and strengthen bonds with family and friends can have a positive impact on their PWB by reducing loneliness [11] and social isolation [48], and strengthening life satisfaction [49]. Based on these findings, we can make this hypothesis;

- H1a: Social media usage among university students is positively related to their PWB

SWB refers to an individual's overall contentment and happiness, taking into account their personal perception of the significance they place on various aspects of their life. Put simply, SWB encompasses a comprehensive assessment of one's life, encompassing both cognitive evaluations of life satisfaction (cognition) and emotional assessments of feelings and moods (emotion) [50]. This concept is a growing area of concern in light of the increase in mental health issues in higher education [51]. A decline in SWB is frequently observed prior to the onset of more severe mental health problems and behavioral issues, including but not limited to depression, suicidal tendencies, and dropping out of college [52, 53]. However, some studies have linked social media usage to better SWB. For instance, prior research has demonstrated that social media platforms like Facebook can contribute to users’ accrual of network social capital, thus bolstering SWB [54]. Also, positive feedback received from individuals with whom one interacts online can significantly enhance overall well-being and mental health. And more frequent quality-based online communication with relatives, friends, family members, and relevant others was also found to have positive impacts on SWB [55] through lowered depression over time [56] and enhanced life satisfaction [55].

Moreover, according to the flow theory, individuals can experience a state of flow when they direct their attention toward accomplishing a specific task or overcoming a challenge in order to attain certain objectives [57]. This state of flow is characterized by a sense of fulfillment, enhanced cognitive abilities, heightened motivation, and overall happiness [58]. That is to say, flow improves people’s SWB. To experience a flow state, three conditions need to be fulfilled: having a clear goal and a perceived challenge, maintaining a balance between the difficulty of the challenge and one's skill level, and receiving immediate feedback on progress. Social media, with its enjoyable and controllable nature, provides these conditions and allows users to have an immersive experience, making it a significant source of flow experiences and contributing to people's SWB. In light of this principle, as students increase their usage of social media, they allocate a greater portion of their focus and energy toward engaging with these platforms. In the process of pursuing their objectives, such as engaging in lively conversations with friends via popular messaging applications like WeChat and QQ, or exhibiting their picturesque travel snapshots on platforms like Weibo, they might unexpectedly receive affirming feedback and positive responses from their virtual connections. This immersive and seamless flow experience not only enables individuals to unwind and experience a heightened sense of contentment but also directly enhances their overall sense of SWB. Along this line, we can propose the following hypothesis;

- H1b: Social media usage by university students is positively associated with their SWB.

### Self-esteem and online social support as mediators

Self-esteem refers to an individual's enduring attitude, whether positive or negative, towards oneself that remains consistent regardless of various circumstances and the passage of time [59, 60]. Self-esteem is crucial, especially for young individuals, as they are going through a period of forming their identity, and feedback about themselves can greatly impact their self-esteem [61]. Research has demonstrated that individuals who possess high self-esteem often experience lower levels of aggressive negative emotions and depression compared to those with low self-esteem [62, 63]. Research also revealed that self-esteem functions as an important and positive predictor of PWB and SWB [64] and success later in life [65]. By contrast, people who have low self-esteem are likely to be socially anxious, shy, lonely, and introverted. Individuals who experience a decrease in their self-esteem frequently limit their interactions with others, which can impede the formation of close and supportive relationships that are crucial for their overall well-being [66]. Additionally, they tend to have less stable and satisfying relationships compared to those with high self-esteem [67]. Furthermore, individuals with low self-esteem tend to engage in self-victimization and shift blame onto others when faced with social failures, rather than acknowledging their own choices. These tendencies lead to avoidance of social interactions, unfamiliar situations, and a general disconnection from society, which in turn heighten the chances of developing social anxiety and depression [68].

However, interacting with others on social media can generate favorable impacts on one's self-esteem when individuals experience a feeling of belonging and receive encouragement and assistance from their online connections. In the study by Apaolaza et al. [69], people socializing on social media sites can experience a rise in self-esteem and improvement in their SWB. Moreover, receiving positive feedback on social media can also help boost self-esteem, as others' responses to an individual's posts are usually positive. Studies have shown that the number of likes on social networking sites like Facebook is linked to higher self-esteem [70]. In more recent research using objective data, it was revealed that Facebook 'likes' have a positive association with happiness, as they boost self-esteem [71]. Similarly, engaging in self-reflection on social media can have a positive effect on one's self-esteem. By allowing users to carefully select and present information about themselves, social media enables individuals to highlight their positive attributes and experiences, which can boost their self-esteem when they review their profile or past interactions with others [40, 72]. As a result, we hypothesized that;

- H2a: There exists a mediating role of self-esteem in the relationship between social media usage by university students and their PWB and SWB.

Social support, being one of the most prominent factors that provide protection, plays a crucial and indispensable role in the prevention of mental illnesses [73, 74]. It serves as a vital element in safeguarding individuals from the onset and development of psychological disorders [75]. When individuals received increased levels of social support, they experienced a decrease in feelings of loneliness and an increase in overall happiness [76]. Online social support refers to the emotional, informational, and instrumental support received through the Internet, as well as the feeling of connection and acceptance from friends, family, and other individuals within one's social circles. Online social support represents the extension of social support that is traditionally available in the physical world to the virtual realm of cyberspace and can enhance the well-being and overall health of individuals, both physically and mentally. This support is facilitated by online platforms and serves as a source of comfort, guidance, and a sense of belonging in times of need. It encompasses various forms of assistance, ranging from empathetic conversations and advice to tangible resources and assistance [77, 78]. Through online social support, individuals are able to seek solace, share their experiences, and build meaningful relationships with others, ultimately enhancing their overall well-being and social connectedness in the digital realm. Past research has indicated that the utilization of mobile social media platforms can effectively fortify individuals' connections with others, thus offering them online social support, which in turn aids in the improvement of their well-being [79, 80]. A recent review by Gilmour et al. [81] discovered that using social networking sites like Facebook for seeking social support can enhance users’ overall well-being, as well as improve both physical and mental health. Additionally, it was found to decrease instances of mental illnesses such as depression, anxiety, and loneliness. Thus, online social support seems to have promising effects on young people’s well-being. Along this line, we made the following hypotheses;

- H2b: There exists a mediating role of online social support in the relationship between social media usage by university students and their PWB and SWB.

In addition, it has been revealed that self-esteem is a crucial individual factor affecting social support [82]. Researchers contend that people having greater self-esteem are more inclined to have positive self-evaluations [83], gain acceptance from others [84], and exhibit proactive and optimistic behaviors in online contexts [85]. As a result, they are more likely to receive social support and assistance from their online communities. In comparison, individuals with lower self-esteem typically have negative opinions about themselves, display more negative behavior online, and may not receive as much social support on the Internet [86]. Furthermore, empirical studies also found a positive relationship between the two variables [87, 88]. Given the literature review, we proposed;

- H2c: University students’ self-esteem is positively related to their online social support.

### Cyberbullying as a moderator

Cyberbullying, according to Rafferty and Vander Ven [88], was depicted as ‘repeated unwanted, hurtful, harassing, and threatening interaction through electronic communication media’. In contrast to conventional websites, social media platforms provide users with the unique opportunity to selectively share information and content by adjusting their account settings. This remarkable feature has granted young individuals an unprecedented level of access to personal information, as well as a readily accessible platform to exploit this information to their advantage when interacting with others. Cyberbullying can manifest itself across various platforms such as text messages, electronic mail, online chat rooms, and social networking sites. It has emerged as a substantial public health worry due to its potential to induce mental and behavioral health complications, along with an elevated susceptibility to suicidal tendencies [89]. In fact, cyberbullying poses a detrimental impact on all groups of people who have access to technology, but its consequences are particularly severe for students due to their vulnerable age and susceptibility to online harassment [90].

According to existing literature, individuals who fall victim to cyberbullying commonly experience a range of psychological issues, including but not limited to stress, depression, feelings of isolation, loneliness, low self-esteem, low academic success, fear of attending school, heightened levels of social anxiety and suicidal ideations [91]. Furthermore, numerous research studies have consistently demonstrated that cyberbullying inflicts severe emotional and physiological harm upon vulnerable individuals who find themselves unable to defend against such attacks [92], decreasing their SWB [93] and causing psychological challenges, such as behavioral issues, alcohol consumption, smoking, and diminished dedication to their academic pursuits [94]. Due to the detrimental impact of cyberbullying on individuals' well-being, it hinders students' academic success as they struggle to overcome the emotional distress caused by this form of harassment. It was revealed that cyberbullying victimization is strongly associated with various psychological issues such as anxiety, depression, substance abuse, diminished self-esteem, interpersonal difficulties, strained familial relationships, and subpar academic performance among university students [95].

Research consistently reveals that individuals who are bullied typically have lower levels of self-esteem compared to those who are not victimized [34, 96]. And empirical studies based on student samples also confirmed that experience of cyberbullying as a victim was found to be correlated with significantly lower levels of self-esteem [94, 97]. In a more recent study based on Chinese university students, Ding et al. [98] also observed a negative association between cyberbullying and self-esteem. On the other hand, cyberbullying often comes in many forms, such as being ignored, disrespected, threatened, made fun of, and harassed, causing psychological and emotional distress for the victim. Such undesirable feelings and experiences may dampen their motivation and weaken their enthusiasm to engage with online communities [35], thus decreasing potential online social support they would receive from peers, friends, family members, educators, and romantic partners. Also, cyberbullying erodes the trust individuals have in their online connections so that they would become more cautious about sharing personal information or expressing their thoughts and feelings online [99], thus hindering the development of genuine connections and limiting the depth of online social support received. In addition, continuous exposure to cyberbullying can damage a person's self-esteem, self-confidence and self-worth, resulting in a wrong belief that they are undeserving of support or that others will not empathize with their experiences [95, 100] which may lead to refraining from seeking or accepting online social support. And those suffering from cyberbullying may also choose not to seek online or offline social support due to fear or anxiety, which would in turn have an adverse impact on their well-being [101].

Based on these findings, it can be inferred that the occurrence of cyberbullying might impact the connection between students' engagement with social media platforms and the positive outcomes it typically fosters. Thus, we hypothesized that;

- H3a: Cyberbullying moderates the relationship between social media usage by university students and their self-esteem, wherein the relationship is weaker when cyberbullying is high.
- H3b: Cyberbullying moderates the relationship between social media usage by university students and their online social support, wherein the relationship is weaker when cyberbullying is high.
- H3c: Cyberbullying moderates the relationship between social media usage by university students and their PWB, wherein the relationship is weaker when cyberbullying is high.
- H3d: Cyberbullying moderates the relationship between social media usage by university students and their SWB, wherein the relationship is weaker when cyberbullying is high.

## Methodology

### Participants and procedure

The data for the present study were collected via an online survey carried out from April 2023 to May 2023. The survey was based on Wenjuanxing (www.wjx.cn), a widely accepted and professional online survey platform for questionnaire design and data collection in China. Questionnaire links can be sent to participants through various social media platforms, such as WeChat, QQ, Weibo, and email. Once the survey is finished, the statistical charts can be downloaded to a Word document for SPSS analysis online, or the original data can be downloaded to Excel and imported into SPSS software for further analysis. It has advantages due to its high efficiency, high quality and low cost. In the present study, questionnaires were designed in Chinese using Wenjuanxing and were then distributed and collected via WeChat and QQ, two popular social platforms that many Chinese people use on a daily basis.

A total of 1,301 active responses were recorded in a response to 1,500 distributed questionnaires (86.73% response rate). Each individual who took part in the research willingly agreed to participate and were given the assurance that their answers would be kept confidential, anonymous, and solely used for the purpose of conducting the study. Since the current study aimed at investigating the influence of social media usage, those who had no access to electronic devices or reported having not used any social media platforms were excluded (*N* = 9). And following careful data cleansing, the final sample comprised 1,004 students, and their major characteristics are displayed in Table 1. The research participants consisted of both undergraduate (825) and graduate students (179) enrolled in 135 universities and colleges throughout China. Of the total participants, 48.11% were female students and 68.92% were from single-child families. The age range of the sample ranged from 18 to 31 years (*M* = 23.78, *SD* = 4.06).

**Table 1:** General demographic characteristics (*N* = 1,004)

Variables	Categories	*N*	Freq (%)
*Gender*	Female	483	48.11
	Male	521	51.89
*Age (years)*	18–22	436	43.43
	23–27	353	35.16
	28–31	215	21.41
*Education level*	Undergraduate	825	82.17
	Graduate	179	17.83
*Single-child family*	Yes	692	68.92
	No	312	31.08
*Family origin*	Urban	609	60.66
	Rural	395	39.34

### Measures

Scale items used in the present study were drawn from the extant literature; thus, well established and validated scales widely applied in prior studies were employed to measure the various constructs in the model shown in Fig. 1. Given that the respondents in the study are Chinese, the English-language scales used for measuring social media usage and cyberbullying were translated into Chinese. To guarantee that the language was consistent in its meaning, a technique known as back-translation designed by Brislin [102] was employed. Specifically, this process involved the translation of items from English to Chinese by a bilingual linguist and the back-translation by another bilingual scholar. The other scales we employed were Chinese versions with valid and reliable psychometric properties.

#### Social media usage scale

In order to assess individuals' engagement on online social platforms, the researchers chose the 9-item general social media usage subscale from the Media and Technology Usage and Attitude Scale (MTUAS) devised by Rosen et al. [103]. The original MTUAS scale was designed to assess technology and media usage as well as attitudes toward technology. It consists of 60 questions, each of which measures 1 of 11 usage subscales of the questionnaire, and the subscales can be applied collectively or separately. Participants were requested to provide information regarding how often they engage in various activities on social media platforms (e.g., “Read postings; Comment on postings, status updates, photos, etc.”). Each participant assessed the accuracy of the statements using a frequency scale that ranged from 1 (*never*) to 10 (*all the time*) with higher scores indicating more social media usage. According to Rosen et al. [103] and Barton et al. [104], the general social media usage scale demonstrated good reliability and validity with the alpha coefficient calculated at 0.97 and 0.90, respectively. In the current study, the measure showed good reliability (Cronbach’s α = 0. 906).

#### Cyberbullying scale

An instrument devised by Ybarra et al. [105] captures the prevalence of an individual experiencing aggressive behavior online across various digital media platforms and electronic devices. The four-item self-report scale assesses the frequency of being subjected to such behaviors within the preceding year on a 5-point Likert scale with response options ranging from 1 (*not sure*) to 5 (*often*). Sample statements include: (a) “Someone made a rude or mean comment to me online”, (b) “Someone sent a text message that said rude or mean things”. Higher scores represent greater levels of cyberbullying as a victim. In the present study, the reliability of the scale calculated based on the current sample was high (Cronbach’s α = 0.818).

#### Self-esteem scale

The Rosenberg Self-Esteem Scale (RSES; Rosenberg, [59]) was adopted to assess global self-esteem with 10 statements on a 4-point Likert scale. This measure has already been translated into Chinese, demonstrating reliable and adequate psychometric properties [85, 106]. Participants’ response categories were set as 1(*strongly disagree*) and 4 (*strongly agree*). Example questions include: (a) “I feel that I have a number of good qualities,” and (b) “I take a positive attitude toward myself.” The five negatively worded items on the scale were reverse scored and the height of the scores taken from the measure suggests that a respondent’s self-esteem is high. For the present study, the measure demonstrated good reliability (Cronbach’s α = 0.945).

#### Online social support scale

The measure of online social support an individual receives was adapted from the Chinese short version of the Online Social Support Scale (OSSS-CS) developed by Zhou and Cheng [107] as this 20-item instrument has been translated into Chinese and has been tested in Chinese populations demonstrating good internal consistency and high construct validity for its four subscales: esteem/emotional support (0.92), social companionship (0.80), informational support (0.98), and instrumental support (0.92). These four factors were also validated based on confirmatory factor analysis (CFA). Example items include: (a) “People encourage me when I am online”, (b) “People help me learn new things when I am online”, and (c) “When I am online, people help me with school or work”. Participants were asked to rate the frequency of social support in these dimensions they received from the online world and their responses were recorded on a 5-point Likert scale with anchors of 1 (*never*) and 5 (*a lot*). Higher scores indicate greater online social support. In the present study, the measure demonstrated good reliability (Cronbach’s α = 0.956).

#### PWB scale

The PWB of the participants was evaluated using a shorter Chinese version for Ryff and Keyes’ [8] PWB Scale [108]. The 18-item scale is broken down into six different facets: autonomy, environmental mastery, personal growth, positive relations with others, purpose in life, and self-acceptance. Each aspect was measured by three items and the response to the individual questions was reverse-coded and configured with a 7-point Likert scale, ranging from 1 (*strongly agree*) to 7 (*strongly disagree*). Example items are: (a) “I tend to be influenced by people with strong opinions,” (b) “I have not experienced many warm and trusting relationships with others," and (c) "In many ways I feel disappointed about my achievements in life." Higher scores mean greater PWB. The shortened version scale has been adopted in a series of previous studies on Chinese samples with good internal consistency [109]. For the current study, the scale was reliable (Cronbach’s α = 0. 959).

#### SWB scale

The revised version of the College Student SWB Questionnaire (CSSWQ) with 16 self-report items that comprise four subscales was adopted to assess participants’ SWB in terms of academic efficacy, college gratitude, school connectedness, and academic satisfaction [53]. The four dimensions were measured using four items, respectively, on a 7-point Likert scale with anchors of 1 (*strongly disagree*) and 7 (*strongly agree*). Sample statements are: (a) “I have had a great academic experience at this college,” (b) “I am a diligent student,” and (c) “I feel thankful for the opportunity to learn so many new things." The overall well-being score was calculated by computing the average of all the items on the scale with higher scores reflecting better SWB. This scale has been translated into Chinese and validated on Chinese samples [110], revealing reliable and valid psychometric properties. In the present study, the measure demonstrated good reliability (Cronbach’s α = 0.953).

### Statistical analysis

Before further analyses, we carried out a confirmatory factor analysis (CFA) using AMOS 26.0 to ensure the validity and reliability of the study variables. The potential common method variance (CMV) was checked considering self-report questionnaire was the principal method for obtaining data. After that, data analysis in the study was carried out in three steps using SPSS 26.0. Firstly, descriptive statistics and Pearson’s correlations were summarized and calculated. Then, to test the proposed hypotheses in the study, we employed Haye’s PROCESS macro Model 6 (version 3.4.1 software) [111] to test the mediating role of self-esteem and online social support in the relationship between social media usage and PWB and SWB. Finally, Haye’s PROCESS macro Model 85 [111] was conducted to test whether the first stage of indirect relationships and the direct association between social media usage and PWB and SWB was moderated by cyberbullying. In the process, all variables were standardized and the interaction terms were computed from the standardized variables. The bias-corrected percentile bootstrap method and 95% confidence intervals (CI) were applied. If the effect does not include 0 in the 95% CI, it is considered to be statistically significant. Moreover, the simple slope analysis was employed to evaluate the moderating effects [112]. We plotted the relationship between the independent variable (social media usage) and the dependent variables (self-esteem and online social support) when the levels of the moderator variable (cyberbullying) were one standard deviation below and one standard deviation above mean value of the moderator variable. In addition, demographic variables (i.e., gender, age, family origin) were controlled during the analyses. A *p*-value of < 0.05 was considered to be statistically significant.

## Results

### Validity, construct reliability, and common method variance

The content validity and reliability of the study variables analyzed through CFA are displayed in Table 2. As shown in the table, the item loadings of all factors in the study exceed the threshold value of 0.60 as recommended by Hair et al. [113]. To ensure the convergent validity of our model, we conducted an analysis of the composite reliability (CR), average variance extracted (AVE), and Cronbach alpha (CA) of all the constructs. The findings from this analysis revealed that the CR and CA values for all the constructs exceeded the recommended threshold of 0.70, indicating a high level of internal consistency. Additionally, construct validity is also confirmed because the AVE values for all the constructs were also above the suggested threshold of 0.50, as advised by previous research studies [114, 115]. To assess the discriminant validity of our study, we employed the methodology suggested by Fornell and Larcker [114]. Our approach involved examining the square root values of AVE for each construct and comparing them with their respective inter-correlations. Considering that the square root of AVE for each factor is greater than its correlations with other factors, it can be concluded that discriminant validity is also established (see Tables 2 and 3 for comparison).

**Table 2:** Results of confirmatory factor analysis (CFA)

Variables	Factor Loadings	CA (*α*)	CR	AVE	Square Root of AVE
Social Media Usage	.670-.807	.906	.907	.520	.721
Cyberbullying	.704-.785	.818	.819	.531	.729
Self-Esteem	.768-.848	.945	.945	.631	.794
Online Social Support	.681-.817	.956	.957	.524	.724
PWB	.690-.842	.959	.959	.564	.751
SWB	.696-.832	.953	.953	.561	.749

*CA* Cronbach’s alpha, *CR* composite reliability, *AVE* average variance extracted

**Table 3:** Means, standard deviation, and correlations

	***M* (*SD*)**	1	2	3	4	5	6	7	8	9	10	11
1.Age	23.78(4.06)	-										
2.Gender	1.52(0.50)	-.00	-									
3.Family origin	1.39(0.49)	-.01	-.09**	-								
4.Family type	1.31(0.46)	-.05	-.05	.20**	-							
5. Education level	1.18(0.38)	-.05	-.02	.15**	.19**	-						
6.SMU	6.54(1.46)	-.01	.02	-.01	-.05	-.06	-					
7.CBB	3.70(0.77)	.00	.02	.01	-.01	-.05	.18**	-				
8.SEE	2.51(0.67)	.01	-.01	-.03	-.02	-.04	.45**	-.18**	-			
9.OSS	3.68(0.72)	-.01	.00	-.05	-.01	-.10**	.43**	-.20**	.62**	-		
10.PWB	3.94(1.13)	.01	.01	-.02	-.01	-.03	.40**	-.27**	.54**	.55**	-	
11.SWB	4.70(1.13)	.02	.03	-.10**	-.03	-.08*	.46**	-.16**	.50**	.53**	.40**	-

*SMU* social media usage, *CBB* cyberbullying, *SEE* self-esteem, *OSS* online social support

^*^ *p* < .05

^**^ *p* < .01

In order to minimize the risk of CMV in our data, we implemented multiple strategies to ensure the accuracy and reliability of the self-reported answers provided by the participants. For instance, as a procedural measure, we took into consideration the suggestions put forward by Podsakoff et al. [116] to address any potential concerns regarding the anonymity and confidentiality of our participants. We took great care in ensuring our participants that their identities would be kept strictly confidential, and that any information they shared would be treated with the highest level of confidentiality. Additionally, we employed the Herman single-factor test, as recommended by Podsakoff et al. [116], to evaluate the potential threat of CMV in our study. The results of this test indicated that the first factor accounted for 33.97% of the variance, suggesting that there is no significant problem of CMV present in our study.

### Preliminary analyses

Descriptive statistics and correlation matrix between the variables are reported in Table 3. As expected, all proposed path variables were revealed to be intercorrelated significantly (see Table 3). Significant positive correlations were obtained between social media usage and PWB (*r* = 0.40,* p* < 0.01) and SWB (*r* = 0.46,* p* < 0.01), respectively with large effect sizes. Self-esteem and online social support were found to be positively associated with social media usage (*r* = 0.45,* p* < 0.01; *r* = 0.43,* p* < 0.01), PWB (*r* = 0.54,* p* < 0.01; *r* = 0.55,* p* < 0.01), and SWB (*r* = 0.50,* p* < 0.01; *r* = 0.53,* p* < 0.01), respectively. In addition, cyberbullying was negatively related to self-esteem (*r* = -0.18,* p* < 0.01), online social support (*r* = -0.20,* p* < 0.01), PWB and SWB (*r* = -0.27,* p* < 0.01; *r* = -0.16,* p* < 0.01), respectively whereas a positive association was observed between this variable and social media usage (*r* = 0.18,* p* < 0.01). In general, no significant relationships were identified between the demographic variables and the other variables under investigation. We, therefore, included them as control variables in the follow-up analyses.

### Testing for the mediating effect

To test the hypothesized relationship between social media usage and outcomes as well as the mediation of self-esteem and online social support, we utilized SPSS PROCESS macros [111]. The results presented in Table 4 revealed that social media usage was positively related to self-esteem (*B* = 0.20, *t* = 15.75, *p* < 0.001), online social support (*B* = 0.09, *t* = 7.00, *p* < 0.001), PWB (*B* = 0.11, *t* = 4.78, *p* < 0.001), and SWB (*B* = 0.19, *t* = 8.36, *p* < 0.001), confirming our hypotheses H1a and H1b. Moreover, the results further showed that self-esteem and online social support mediate the relationship between students’ usage of social media and their PWB and SWB. Specifically, social media usage was significantly and positively associated with PWB via self-esteem (indirect effect = 0.100, *SE* = 0.01, 95% *CI* = [0.075, 0.126]), via online social support (indirect effect = 0.046, *SE* = 0.01, 95% *CI* = [0.030, 0.063]), and via self-esteem and online social support (indirect effect = 0.058, *SE* = 0.01, 95% *CI* = [0.043, 0.074]). Similarly, the utilization of social media by students was also significantly and positively related to their SWB via self-esteem (indirect effect = 0.072, *SE* = 0.02, 95% *CI* = [0.049, 0.097]), online social support (indirect effect = 0.043, *SE* = 0.01, 95% *CI* = [0.027, 0.061]), and the two mediators (indirect effect = 0.054, *SE* = 0.01, 95% *CI* = [0.039, 0.070]). Thus, self-esteem and online social support acted as effective mediators in the association between social media usage and PWB and SWB, supporting H2a, H2b. Moreover, self-esteem had a significant and positive effect on online social support (*B* = 0.57, *t* = 19.76, *p* < 0.001), thus confirming H2c.

**Table 4:** Testing the mediating effect of social media usage on PWB and SWB via self-esteem and online social support

Independent variables	**Mediators**										**Dependent variables**									
	SEE					OSS					PWB					SWB				
	*B*	*SE*	*t*	95%CI		*B*	*SE*	*t*	95%CI		*B*	*SE*	*t*	95%CI		*B*	*SE*	*t*	95%CI	
				LLCI	ULCI				LLCI	ULCI				LLCI	ULCI				LLCI	ULCI
Constant	1.21	.18	6.70	.86	1.56	1.87	.17	11.13	1.55	2.19	.03	.29	.11	-.51	.58	.98	.29	3.34	.44	1.54
SMU	.20	.01	15.75	.18	.23	.09	.01	7.00	.07	.12	.11	.02	4.78	.06	.15	.19	.02	8.36	.14	.23
SEE						.57	.03	19.76	.52	.63	.49	.06	8.77	.38	.60	.35	.06	6.29	.24	.46
OSS											.49	.05	9.51	.39	.59	.46	.05	8.83	.35	.57
*R*^*2*^	.20					.42					.38					.22				
*F*	41.85***					103.36***					76.64***					47.72***				
Indirect effects via specific mediator(s)											Effect	SE		95%CI		Effect	SE		95%CI	
														LLCI	ULCI				LLCI	ULCI
Indirect effect of SMU via SEE											.100	.01		.075	.126	.072	.02		.049	.097
Indirect effect of SMU via OSS											.046	.01		.030	.063	.043	.01		.027	.061
Indirect effect of SMU via SEE and OSS											.058	.01		.043	.074	.054	.01		.039	.070

Unstandardized regression coefficients are presented; Bootstrap sample size = 5000, *LLCI* Bias corrected lower limit confidence interval, *ULCI* Bias corrected upper limit confidence interval

^*^p < .05

^**^p < .01

^***^p < .001

### Testing for moderated mediation

In Hypothesis 3, cyberbullying was projected to moderate the first phase of the indirect associations as well as the direct relations between social media usage and PWB and SWB. To test these hypotheses, we performed a moderated mediation analysis by using Haye’s PROCESS macro [111] in SPSS and investigated Cyberbullying across the levels. Concerning the relationships among study variables, as shown in Table 5, cyberbullying was negatively correlated with self-esteem (*B* = -0.24, *t* = -10.24, *p* < 0.001), online social support (*B* = -0.16, *t* = -7.16, *p* < 0.001), PWB (*B* = -0.30, *t* = -7.67, *p* < 0.001), and SWB (*B* = -0.19, *t* = -4.67, *p* < 0.001). The effect of social media usage on self-esteem (*B* = 0.22, *t* = 17.69, *p* < 0.001) and online social support (*B* = 0.12, *t* = 9.12, *p* < 0.001) was significant, and more importantly, this effect was moderated by cyberbullying (*B* = -0.11, *t* = -7.30, *p* < 0.001; *B* = -0.10, *t* = -6.66, *p* < 0.001), respectively. Contrary to our H3c and H3d, the direct relationships between social media usage and PWB (*B* = 0.00, *t* = 0.10, *p* > 0.05) and SWB (*B* = 0.00, *t* = 0.11, *p* > 0.05) were not significantly moderated by cyberbullying. Furthermore, the bias-corrected percentile bootstrapping results revealed that the indirect effect of social media usage on PWB via self-esteem (Index of moderated mediation = -0.05, *SE* = 0.01, 95% *CI* = [-0.07, -0.03]) and online social support (Index = -0.04, *SE* = 0.01, 95% *CI* = [.-0.06, -0.03]) was moderated by cyberbullying. Likewise, the relationship between social media usage and SWB was indirect and moderated by cyberbullying via self-esteem (Index = -0.04, *SE* = 0.01, 95% *CI* = [-0.05, -0.02]) and online social support (Index = -0.04, *SE* = 0.01, 95% *CI* = [-0.06, -0.03]). In addition, results showed that the indirect effects of social media usage by students via self-esteem on their PWB (effect = 0.056, *SE* = 0.01, 95% *CI* = [0.036, 0.078]) and SWB (effect = 0.041, *SE* = 0.01, 95% *CI* = [0.024, 0.061]) were weaker at + 1SD than at -1SD (effect = 0.128, *SE* = 0.02, 95% *CI* = [0.093, 0.165]; effect = 0.094, *SE* = 0.02, 95% *CI* = [0.061, 0.130]), respectively. Also, a similar pattern was observed for the indirect effects of social media usage via online social support on PWB (effect = 0.019, *SE* = 0.01, 95% *CI* = [0.003, 0.036]) and SWB (effect = 0.019, *SE* = 0.01, 95% *CI* = [0.003, 0.037]) at higher level of cyberbullying than at lower level (effect = 0.082, *SE* = 0.01, 95% *CI* = [0.058, 0.107]; effect = 0.081, *SE* = 0.01, 95% *CI* = [0.055, 0.107]), respectively. These results have given support to our H3a and H3b.

**Table 5:** Testing the moderated mediation model of social media usage on PWB and SWB

Independent variables	Mediators										Dependent variables									
	SEE					OSS					PWB					SWB				
	*B*	*SE*	*t*	95%CI		*B*	*SE*	*t*	95%CI		*B*	*SE*	*t*	95%CI		*B*	*SE*	*t*	95%CI	
				LLCI	ULCI				LLCI	ULCI				LLCI	ULCI				LLCI	ULCI
Constant	2.55	.15	17.47	2.27	2.84	2.74	.16	17.48	2.43	3.05	.1.21	.30	4.06	.63	1.79	2.49	.30	8.20	1.90	3.09
SMU	.22	.01	17.69	.19	.24	.12	.01	9.12	.10	.15	.16	.02	7.17	.12	.21	.22	.02	9.49	.18	.27
SEE						.47	.03	15.73	.41	.53	.42	.06	7.64	.31	.53	.31	.06	5.50	.20	.42
OSS											.41	.05	7.88	.31	.52	.41	.05	7.65	.31	.52
CBB	-.24	.02	-10.24	-.28	-.19	-.16	.02	-7.16	-.21	-.12	-.30	.04	-7.67	-.38	-.22	-.19	.04	-4.67	-.26	-.11
SMU*CBB	-.11	.02	-7.30	-.14	-.08	-.10	.01	-6.66	-.13	-.07	.00	.02	.10	-.05	.05	.00	.03	.11	-.05	.05
*R*^*2*^	.31					.47					.42					.39				
*F*	55.65***					97.11 ***					70.89***					63.68***				
Conditional indirect effects at a specific value of moderator											Effect	SE	95%CI			Effect	SE	95%CI		
													LLCI	ULCI				LLCI	ULCI	
Indirect effect of SMU via SEE								-1 (low)			.128	.02	.093	.165		.094	.02	.061	.130	
								+1(high)			.056	.01	.036	.078		.041	.01	.024	.061	
Indirect effect of SMU via OSS								-1 (low)			.082	.01	.058	.107		.081	.01	.055	.107	
								+1(high)			.019	.01	.003	.036		.019	.01	.003	.037	

Unstandardized regression coefficients are presented

^*^p < .05

^**^p < .01

^***^p < .001

**Fig. 2:**
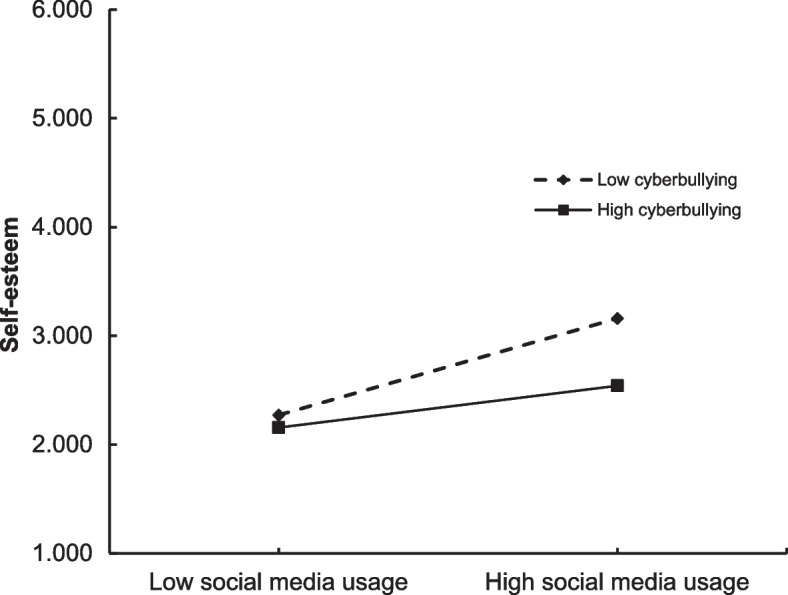
Cyberbullying moderates the relationship between social media usage and self-esteem

**Fig. 3:**
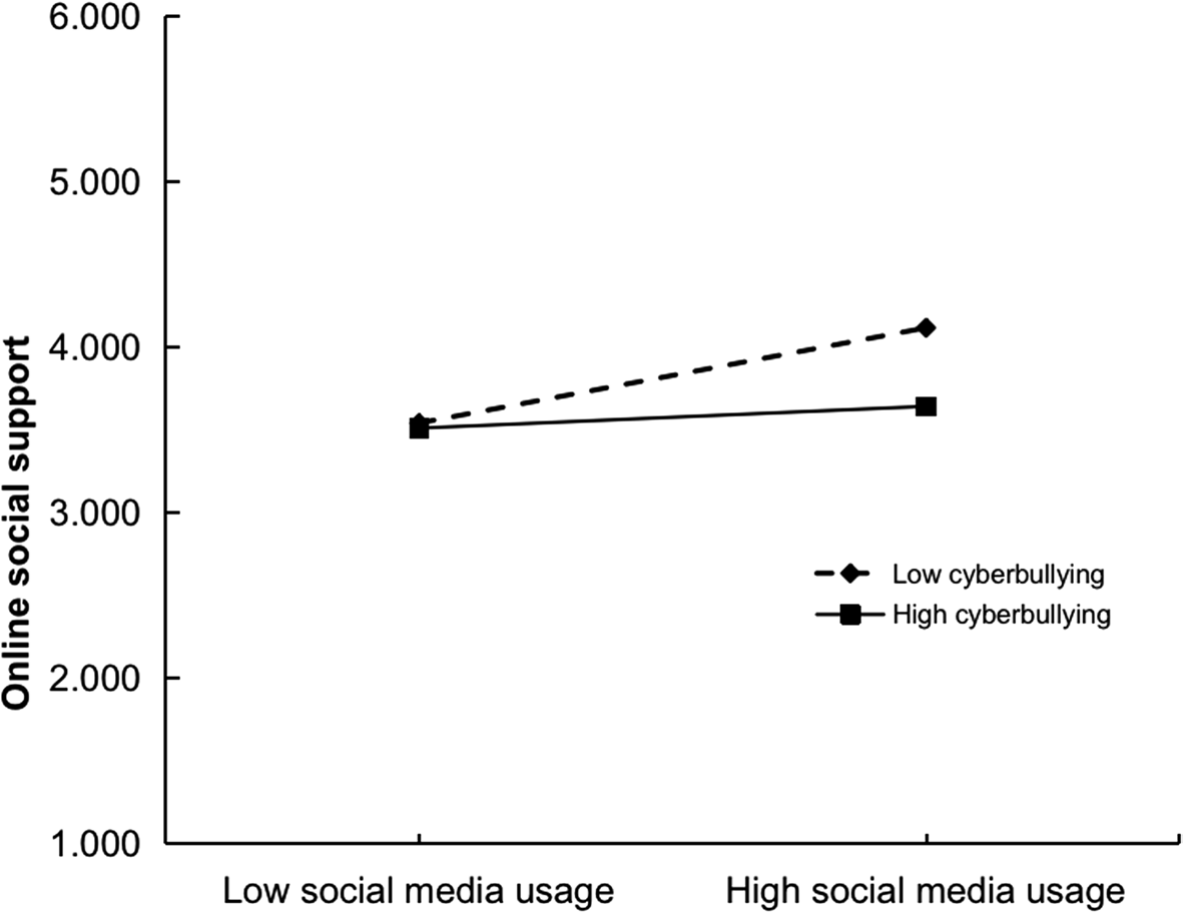
Cyberbullying moderates the relationship between social media usage and online social support

For clarity, we also plotted graphical diagrams to better examine the role of cyberbullying as a moderator in the relations between social media usage and self-esteem (Fig. 2) and online social support (Fig. 3), separately for students experiencing low and high cyberbullying (at 1 *SD* below the mean and 1 *SD* above the mean, respectively). Simple slope tests suggested that the relationships between social media usage and self-esteem and online social support were statistically weaker respectively when at the higher level of cyberbullying.

## Discussion

In this study, a moderated mediation model was formulated to explore whether students’ utilization of social media would be indirectly associated with their PWB and SWB via self-esteem and online social support and whether the first phase of this indirect relationship and the direct correlation would be moderated by cyberbullying they have experienced. Although numerous studies have examined the impacts of social media usage among various groups of people, especially children, this study is one of the few that considers both PWB and SWB as outcome variables among Chinese university students, a sample that has been insufficiently examined. Moreover, this study provides a probable explanation as to why university students' frequent use of social media results in higher levels of PWB and SWB. Moreover, it is the first empirical study confirming the mediating roles of self-esteem and online social support underlying this linkage. The research findings further our understanding of how social media usage impacts users’ well-being and what role cyberbullying plays in the process.

Consistent with our expectations, social media usage by university students positively predicted their PWB and SWB; and self-esteem and online social support mediated the relationships, which extends previous theoretical and empirical studies. Specifically, it helps advance our understanding of the intricate relationship between social media usage and people’s well-being, especially PWB and SWB. Previous research on this association has generated varied results. Some studies have observed a negative relationship while others have acknowledged that a positive association exists as social media can facilitate online social connections [117] and reduce the levels of negative emotions and feelings, such as stress, loneliness, depression, and the sense of social isolation [48], thus beneficial to users’ PWB. The research findings suggest that incorporating social media into the daily lives of college students and actively engaging with shared content can have a profound impact on their self-esteem and access to diverse forms of online social support, which, in turn, has the potential to enhance their overall PWB and SWB. In previous empirical studies [118, 119], self-esteem was mainly found to be positively correlated with several indicators of SWB including affect, meaning in life, and subjective vitality. The present study contributes to the existing body of research by specifically identifying the positive associations between self-esteem and both PWB and SWB in relation to the usage of social media platforms. In this competitive world, healthy self-esteem is required for university students to effectively deal with potential psychological distress that may arise in their academic and career pursuits. And in accordance with self-affirmation theory, greater self-esteem can work as a buffer against unpleasant and stressful experiences and failures [120]. Furthermore, Sociometer Theory [121] suggests that an individual's self-esteem is influenced by their sense of social acceptance and the importance placed on their relationships. This theory provides further insight into the strong correlation between self-esteem and PWB. In collectivistic cultures like China, where social bonds are highly valued, young adults place a great emphasis on their connections with others, particularly within their families and interpersonal relationships. As a result, individuals with higher levels of self-esteem are more likely to experience greater PWB, as their self-esteem serves as a potential indicator of their value within their social circles. In addition to self-esteem, our study also identified positive effects of online social support on students’ well-being consistent with prior research [122]. The reason behind this phenomenon can be attributed to the fact that students who have a vast network of connections on social media and dedicate a considerable amount of time to actively engaging in various interactions on these platforms are more likely to garner a substantial amount of support from their online acquaintances [123]. As the number of friends a user possesses increases, the probability of receiving positive and supportive comments on their status updates, appreciation for their uploaded photos, and congratulations for their personal accomplishments also increases. This correlation implies that a larger social circle enhances the likelihood of receiving encouragement and validation from friends. This particular positive experience, which is frequently absent in face-to-face interactions, can strengthen the feeling of being a part of a social network and instill a sense of being valued, respected, and esteemed among students. As a result, it can lead to the development of a positive psychological and emotional state, ultimately contributing to an elevated level of SWB [124].

Apart from the general mediation effect, it is important to highlight the significance of each individual stage within the mediation process. First, our research finding is in line with prior reports that social media usage increases users' self-esteem [69, 70]. Previous research on self-esteem theories has identified a close relationship between the use of various social media sites such as Facebook, Twitter, and Instagram and users’ self-esteem [125, 126], revealing that peer interaction and feedback on the self represents critical predictors of young adults’ self-esteem [127]. In addition to facilitating instant messaging and enabling activities like posting and commenting on photos, social media platforms offer a valuable channel for young people to receive feedback, interact with their peers, enhance their social skills, and gain insights by observing others [79]. College students in China use similar sites like WeChat and Weibo to portray a different version of themselves online by sharing their photos, videos, and other posts within their friend circles or beyond. The likes they receive on social media sites are regarded as verification for acceptance and approval within their groups of peers, which may, in turn, boost their self-esteem. Since the main objective of social media platforms is to encourage communication and connections between individuals, students who frequently use these sites will have a higher likelihood of actively engaging with their fellow peers and more opportunities to receive positive feedback on social network profiles compared to those who use social media less frequently, thus enhancing their self-esteem. And as predicted, students’ higher self-esteem predicted greater online social support, corresponding to research findings by Jin et al. [87] and Zheng et al. [82]. These findings align with the principles of Sociometer Theory [84], which suggests that there is a strong relationship between self-esteem and how individuals perceive acceptance from society and others. People with high self-esteem often feel valued, which in turn encourages them to engage in positive online communication, receive more affirmation and praise from others, and ultimately be accepted within online communities. On the contrary, individuals who possess low self-esteem often harbor a pessimistic outlook towards their own self-image, leading to more negative online interactions and making it harder for them to receive acceptance from online communities, thus hindering their ability to develop a robust online social support system [128].

Furthermore, in line with previous research [79, 80], our findings indicate that there is a positive correlation between the amount of time students spend on social media and the level of online social support they receive or perceive online. Social support in an online setting has attracted the attention of scholars who have studied its prevalence within social networks. One example of this is when individuals show support for their peers by sharing or forwarding online news articles that would be beneficial to their friends in the digital realm. Moreover, public officials have also recognized the significance of social media in providing updates to citizens during critical events such as natural disasters, criminal incidents, or accidents. In such cases, these officials utilize their social media accounts to keep the public informed and engaged. Additionally, people are able to obtain interpersonal support by connecting and interacting with like-minded individuals on various social media platforms. This form of support, commonly referred to as peer support, serves as a valuable resource for college students seeking understanding, guidance, and empathy from others who share similar interests or experiences [129]. Moreover, a previous research study conducted on college students found that when seeking social support, students were more inclined to rely on social media platforms rather than seeking help from their parents or mental health professionals. Many of them believed that social media use provided them with positive experiences, offering a support network and helping them feel more connected with their friends. Additionally, the study indicated that students tended to gravitate towards communities composed of their peers who shared similar interests, such as fandom communities [130]. Building upon a series of similar findings, our study provides new empirical support for the positive effect of social media usage on online social support.

Meanwhile, we identified cyberbullying as a boundary condition variable in our research model. Specifically, the results indicated that the links between social media usage and their PWB and SWB via the two mediators: self-esteem and online social support were weaker for those students suffering greater levels of cyberbullying. In today's technologically advanced society, the issue of online bullying has become a prominent worry in numerous settings. The research we conducted has provided evidence that cyberbullying has the potential to diminish the positive effects that students typically derive from their use of social media. For individuals experiencing a low level of cyberbullying, self-esteem, and online social support can have significant beneficial effects on their PWB and SWB. Increased cyberbullying, however, leads to more psychological distress, reduced life satisfaction, increased depressive symptoms and anxiety [131], or even suicidal thoughts and attempts [132]. However, contrary to part of our hypotheses, cyberbullying did not moderate the direct relationship between social media usage and PWB and SWB. A probable explanation for this is that the relationship between social media usage, cyberbullying, and well-being is multifaceted and influenced by various factors. It is possible that other variables not considered in this study could be influencing these relationships. For instance, as evidenced by previous research [25], cultural and contextual factors like collectivism in Chinese culture can play an important role in the effects of media use on well-being. Meanwhile, as suggested by the Differential Susceptibility to Media Effects Model [133] and Cultivation Theory [134], sociocultural and psycho-demographic factors can also moderate social media effects by strengthening, diminishing, and/or moderating individuals’ cognitive, emotional, and behavioral responses to media. Another possible reason is that individuals affected by cyberbullying might have developed coping strategies or mechanisms (e.g., emotion-focused coping and avoidance-coping) to deal with cyberbullying to lessen its impact on their PWB and SWB [135]. These coping mechanisms might mitigate the expected moderating effect.

### Limitations and future directions

The present investigation provides a more comprehensive insight into the intricate relationship between social media usage by Chinese university students and their PWB and SWB and how such relationship is mediated by self-esteem and online social support, and moderated by cyberbullying. However, several limitations should be taken into consideration when analyzing and interpreting the research findings.

First, in our study, we employed a cross-sectional research design, which is not without its limitations, particularly the potential for common method variance (CMV). To address this concern, we implemented various measures, such as guaranteeing the confidentiality and anonymity of participants and conducting statistical analyses to confirm the absence of CMV. Nonetheless, we recognize that our model's credibility and validity could be further strengthened by employing a longitudinal research design or carrying out an experimental laboratory study. Second, it is important to approach the generalizability of the present findings with caution. It remains uncertain whether the findings in our study based on samples collected from Chinese universities can be applied to samples obtained in different contexts, populations (e.g., children, older adults), and countries. Therefore, more studies are warranted to examine these relationships in more diverse samples and contexts since it is noteworthy that social network sites may have different effects on individuals of different ages or nationalities. Third, given our failure to confirm hypotheses regarding cyberbullying moderating the impact of social media usage on PWB and SWB due to possible deficiencies in our research design, it is important to note that future studies should formulate a more comprehensive research design by taking into account a broader context and more factors (e.g., coping strategies, social contexts, cultural norms, and psycho-demographic factors) that may moderate social media impact on health outcomes. Meanwhile, given that some studies have found negative effects of excessive and problematic use of social media on users’ well-being, it is necessary for future studies to examine specific factors resulting in such detrimental outcomes, such as time spent on social media, active or passive social media use [136], and users’ motives [137]. Third, the current study found support for the important roles of self-esteem and online social support in explaining why social media usage can be beneficial to users’ PWB and SWB, yet some other factors may also take effect. A more extensive investigation is required in order to gain a comprehensive understanding of the specific circumstances under which predictor variables become significant and the ways in which they interact with online processes and individuals' overall well-being, such as positive and negative emotions while using various social networking sites, bridging and bonding social capital, social connectedness, social comparison, and interpersonal competence. In addition, more studies are needed to determine the circumstances in which social media usage can have positive effects, such as investigating whether social networking platforms that encourage more direct social interaction can improve well-being. Furthermore, future studies can also compare the different roles of direct contact and online contact via different social media platforms in affecting people’s overall well-being. Additionally, it could be further explored how previous experiences with specific social media platforms, potentially influenced by the age of the site and the user, impact the association between usage and PWB and SWB.

### Theoretical and practical implications

Despite the limitations, this research has a series of important theoretical and practical implications. First, the current study is one of the few attempts to examine the impact of social media on well-being from both the hedonic and eudaimonic perspectives among university students in the context of China, contributing to the existing literature by empirically confirming the positive implications of social media usage on PWB and SWB. Second, this study extends the extant literature on social media by identifying a mediation pathway that includes self-esteem and online social support, underlying their positive effects. This finding helps shed light on how self-esteem within the theoretical context of Identity Theory and Sociometer Theory can be applied in the digital domain, opening up a new research trajectory to further exploring the effect of various dimensions of self-esteem on health outcomes within the framework of social media research. Also, the examination of online social support as a mediator aligns with communication and media theories that emphasize the importance of technology-mediated communication in shaping relationships and well-being. Moreover, it provides firm support for the Social Compensation hypothesis, which is concerned with how online interaction can generate a host of benefits for individuals struggling with face-to-face interaction due to lack of social skills or low well-being [133], especially during the pandemic. This can enrich our understanding of how these theories apply within a non-WEIRD cultural context, particularly considering the moderating role of cyberbullying. Lastly, another important contribution of our research is the investigation of the moderating role of cyberbullying, which was found to harm the positive utility of social media on students’ PWB and SWB via diminishing the beneficial effects of self-esteem and online social support. This serves as the core theoretical contribution of this study, adding to the previous body of literature on cyberbullying research, especially its moderating role.

In terms of practical contributions, our results highlight the importance and the beneficial outcomes of social media among college students on their overall well-being. This suggests that educational institutions, teachers, administrators, and parents should recognize the positive application of various social media platforms in academia and encourage rational social media use inside and outside schools. Then the positive effects of self-esteem and online social support indicate that students should communicate and interact more frequently with peers, friends, families and important others as a way to increase their self-esteem and seek more emotional and informational support as well as social companionship. However, the finding that cyberbullying victimization as a moderator can reduce the positive effects of social media usage on health outcomes through mediators of self-esteem and online social support indicates that it is important to empower students at-risk for cyberbullying victimization through prevention efforts. Self-esteem as a social construct is especially influenced by interactions with peers. Hence, it is crucial to offer opportunities for cyberbullying victims to connect with their peers, establish strong relationships, and develop meaningful friendships that contribute to their self-worth and foster a positive self-perception. In addition, as for those enduring cyberbullying-related psychological or behavioral problems (e.g., depression, anxiety, social isolation, and suicidal attempts), most Chinese university counselling centers could open online platforms for psychoeducation like training sessions and courses easily accessible through popular apps, such as WeChat and Tencent [138], and offer timely and target psychological interventions and counseling. Most importantly, given the prevalence of cyberbullying in China, it is imperative that universities initiate training programs and provide relevant curricula to empower students with basic skills and knowledge to recognize, prevent, and cope with cyberbullying. Bullying tracking software and similar practices can be utilized to prevent cyberbullying while using social media for academic purposes. The authorities may also implement more stringent laws and regulations against cyberbullying and online harassment to create a safe online environment.

## Data Availability

The datasets used and/or analyzed during the current study are available from the corresponding author on reasonable request.

## References

[1] Leong L-Y, Hew T-S, Ooi K-B, Lee V-H, Hew J-J (2019). A hybrid SEM-neural network analysis of social media addiction. Expert Syst Appl.

[2] Ostic D, Qalati SA, Barbosa B, Shah SMM, Galvan Vela E, Herzallah AM, Liu F (2021). Effects of social media use on psychological well-being: a mediated model. Front Psychol.

[3] Bayer JB, Triệu P, Ellison NB (2020). Social media elements, ecologies, and effects. Annu Rev Psychol.

[4] Twenge JM, Campbell WK (2019). Media use is linked to lower psychological well-being: evidence from three datasets. Psychiatry Q.

[5] Ryan RM, Deci EL (2001). On happiness and human potentials: a review of research on hedonic and eudaimonic well-being. Annu Rev Psychol.

[6] Kahneman, D., Diener, E., & Schwarz, N. (Eds.). Well-being: The foundations of hedonic psychology. Russell Sage Foundation. 1999

[7] Ryff CD (1989). Beyond Ponce de Leon and life satisfaction: new directions in quest of successful ageing. Int J Behav Dev.

[8] Ryff CD, Keyes CL (1995). The structure of psychological well-being revisited. J Pers Soc Psychol.

[9] Diener, E. (Ed.). The science of well-being: The collected works of Ed Diener. Springer Science + Business Media.2009. 10.1007/978-90-481-2350-6

[10] Awad F, Mayasari R (2015). Subjective well-being, psychological well-being, and islamic religiosity. Int J Sci Res (IJSR).

[11] Halston A, Iwamoto D, Junker M, Chun H (2019). Social media and loneliness. Int J Psychol Stud.

[12] Dalvi-Esfahani M, Niknafs A, Kuss DJ, Nilashi M, Afrough S (2019). Social media addiction: applying the DEMA℡ approach. Telematics Informatics.

[13] Jiao Y, Jo M-S, Sarigöllü E (2017). Social value and content value in social media: two paths to psychological well-being. J Organ Comput Electron Commer.

[14] Kim JY, Chung N, Ahn KM (2014). Why people use social networking services in Korea: the mediating role of self-disclosure on subjective well-being. Inf Dev.

[15] Zsila Á, Reyes MES (2023). Pros & cons: impacts of social media on mental health. BMC Psychology.

[16] Wei L, Gao F (2017). Social media, social integration and subjective well-being among new urban migrants in China. Telematics Inform.

[17] Jimenez, Y., & Morreale, P. Social Media Use and Impact on Interpersonal Communication. In C. Stephanidis (Ed.), HCI International 2015—Posters’ Extended Abstracts, 2015; (pp. 91–96). Springer International Publishing.

[18] Chotpitayasunondh V, Douglas KM (2016). How, “phubbing” becomes the norm: the antecedents and consequences of snubbing via smartphone. Comput Hum Behav.

[19] Swar B, Hameed T (2017). Fear of missing out, social media engagement smartphone addiction and distraction: moderating role of self-help mobile apps-based interventions in the youth. Int Conference Health Informatics.

[20] Roberts JA, David ME (2020). The social media party: Fear of missing out (FoMO), social media intensity, connection, and well-being. Int J Human-Computer Interaction.

[21] Vannucci A, Flannery KM, Ohannessian CM (2017). Social media use and anxiety in emerging adults. J Affect Disord.

[22] Kim Y, Lee M (2023). Does social media use mitigate or exacerbate loneliness among korean older adults? focusing on the moderating role of media literacy. Soc Med + Soc.

[23] Dhir A, Yossatorn Y, Kaur P, Chen S (2018). Online social media fatigue and psychological well-being—a study of compulsive use, fear of missing out, fatigue, anxiety and depression. Int J Inf Manage.

[24] Chi LC, Tang TC, Tang E (2022). The phubbing phenomenon: a cross-sectional study on the relationships among social media addiction, fear of missing out, personality traits, and phubbing behavior. Curr Psychol.

[25] Gong J, Firdaus A, Said F, Ali I, Danaee M, Xu J (2022). Pathways linking media use to wellbeing during the COVID-19 pandemic: a mediated moderation study. Soc Med + Soc.

[26] Gong J, Zanuddin H, Hou W, Xu J (2022). Media attention, dependency, self-efficacy, and prosocial behaviours during the outbreak of COVID-19: a constructive journalism perspective. Global Med China.

[27] Cole DA, Nick EA, Zelkowitz RL, Roeder KM, Spinelli T (2017). Online social support for young people: does it recapitulate in-person social support; can it help?. Comput Hum Behav.

[28] Chen Y, Gao Q (2023). Effects of social media self-efficacy on informational use, loneliness, and self-esteem of older adults. Int J Human-Computer Int.

[29] Haslam DM, Tee A, Baker S (2017). The use of social media as a mechanism of social support in parents. J Child Fam Stud.

[30] Li LW, Liang J (2007). Social exchanges and subjective well-being among older Chinese: does age make a difference?. Psychol Aging.

[31] Brochado S, Soares S, Fraga S (2017). A scoping review on studies of cyberbullying prevalence among adolescents. Trauma Violence Abuse.

[32] van Geel M, Vedder P (2020). Does cyberbullying predict internalizing problems and conduct problems when controlled for traditional bullying?. Scand J Psychol.

[33] Carvalho M, Branquinho C, Matos M (2021). Cyberbullying and bullying: impact on psychological symptoms and well-being. Child Indicators Res.

[34] Wachs S, Vazsonyi AT, Wright MF, KsinanJiskrova G (2020). Cross-national associations among cyberbullying victimization, self-esteem, and internet addiction: direct and indirect effects of alexithymia. Front Psychol.

[35] Hsieh Y-P (2020). Parental psychological control and adolescent cyberbullying victimization and perpetration: The mediating roles of avoidance motivation and revenge motivation. Asia Pacific J Soc Work.

[36] Kwan I, Dickson K, Richardson M, MacDowall W, Burchett H, Stansfield C, Brunton G, Sutcliffe K, Thomas J (2020). Cyberbullying and children and young people's mental health: a systematic map of systematic reviews. Cyberpsychol Behav Soc Netw.

[37] Auxier B, Anderson M. Social Media USE in 2021. 2021. Available online at: https://www.pewresearch.org/internet/2021/04/07/social-media-use-in-2021/

[38] Gulzar MA, Ahmad M, Hassan M, Rasheed MI (2022). How social media use is related to student engagement and creativity: Investigating through the lens of intrinsic motivation. Behav Information Technol.

[39] Wu H-Y, Chiou A-F (2020). Social media usage, social support, intergenerational relationships, and depressive symptoms among older adults. Geriatr Nurs.

[40] Cingel DP, Carter MC, Krause H-V (2022). Social media and self-esteem. Curr Opinion Psychol.

[41] Wirtz D, Tucker A, Briggs C, Schoemann A (2021). How and why social media affect subjective well-being: multi-site use and social comparison as predictors of change across time. J Happiness Stud.

[42] Ye S, Ho KKW, Wakabayashi K, Kato Y (2023). Relationship between university students' emotional expression on tweets and subjective well-being: considering the effects of their self-presentation and online communication skills. BMC Public Health.

[43] Chen YA, Fan T, Toma CL, Scherr S (2022). International students’ psychosocial well-being and social media use at the onset of the COVID-19 pandemic: a latent profile analysis. Comput Human Behav.

[44] Yimer BL (2023). Social media usage, psychosocial well-being and academic performance. Community Health Equity Res Policy.

[45] Diener E, Seligman MEP (2004). Beyond money: toward an economy of well-being. Psycholog Sci Pub Interest.

[46] Shaheen MA (2008). Use of social networks and information seeking behavior of students during political crises in Pakistan: a case study. The Intern Information Library Rev.

[47] Shah S, Hussain K, Aftab A, Rizve R (2021). Social media usage and students’ psychological well-being: an empirical analysis of District Mirpur, AJ&K, Pakistan. New Educ Rev.

[48] O’keeffe GS, Clarke-Pearson K, Council on Communications and Media (2011). The impact of social media on children, adolescents, and families. Pediatrics.

[49] Zhan L, Sun Y, Wang N, Zhang X (2016). Understanding the influence of social media on people’s life satisfaction through two competing explanatory mechanisms. Aslib J Inf Manag.

[50] McGillivray, M. Human Well-being: Issues, Concepts and Measures. In: McGillivray, M. (eds) Human Well-Being. Studies in Development Economics and Policy. Palgrave Macmillan, London. 2007.10.1057/9780230625600_1

[51] Twenge JM, Joiner TE, Rogers ML, Martin GN (2018). Increases in depressive symptoms, suicide-related outcomes, and suicide rates among U.S. Adolescents after 2010 and links to increased new media screen time. Clin Psycholog Sci.

[52] Keyes CL, Dhingra SS, Simoes EJ (2010). Change in level of positive mental health as a predictor of future risk of mental illness. Am J Public Health.

[53] Renshaw TL (2018). Psychometrics of the revised college student subjective well-being questionnaire. Can J Sch Psychol.

[54] Wu M-S (2023). The effects of facebook use on network social capital and subjective well-being: a generational cohort analysis from the Taiwan social change survey. Heliyon.

[55] Dienlin T, Masur PK, Trepte S (2017). Reinforcement or displacement? The reciprocity of FtF, IM, and SNS communication and their effects on loneliness and life satisfaction. J Comput-Mediat Commun.

[56] Moukalled SH, Bickham DS, Rich M (2021). Examining the associations between online interactions and momentary affect in depressed adolescents. Front Human Dynamics.

[57] Csikszentmihalyi M (2008). Flow: The psychology of optimal experience.

[58] Moneta GB, Csikszentmihalyi M (1996). The effect of perceived challenges and skills on the quality of subjective experience. J Pers.

[59] Rosenberg M (1965). Society and the adolescent self-image.

[60] Brown, J. D., & Marshall, M. A. The Three Faces of Self-Esteem. In M. H. Kernis (Ed.), Self-esteem issues and answers: A sourcebook of current perspectives, 2006; (pp. 4–9). Psychology Press.

[61] Valkenburg PM, Koutamanis M, Vossen HGM (2017). The concurrent and longitudinal relationships between adolescents' use of social network sites and their social self-esteem. Comput Hum Behav.

[62] Bandura A (1977). Self-efficacy: toward a unifying theory of behavioral change. Psychol Rev.

[63] Sowislo JF, Orth U (2013). Does low self-esteem predict depression and anxiety? A meta-analysis of longitudinal studies. Psychol Bull.

[64] ciçek I (2021). Mediating role of self-esteem in the association between loneliness and psychological and subjective well-being in University students. Intern J Contemporary Educ Res.

[65] Orth U, Robins RW (2014). The development of self-esteem. Curr Dir Psychol Sci.

[66] Fatima M, Niazi S, Ghayas S (2017). Relationship between self-esteem and social anxiety: role of social connectedness as a mediator. Pakistan J Soc Clin Psychol.

[67] Pineiro, Carly Renee, "Social media use and self-esteem in undergraduate students". Theses and Dissertations. 2016;1484. https://rdw.rowan.edu/etd/1484

[68] Tracy JL, Robins RW (2003). "Death of a (Narcissistic) salesman:" an integrative model of fragile self-esteem: comment. Psychol Inq.

[69] Apaolaza V, Hartmann P, Medina E, Barrutia JM, Echebarria C (2013). The relationship between socializing on the Spanish online networking site Tuenti and teenagers’ subjective wellbeings: the roles of self-esteem and loneliness. Comput Hum Behav.

[70] Burrow AL, Rainone N (2017). How many likes did I get?: Purpose moderates links between positive social media feedback and self-esteem. J Exp Soc Psychol.

[71] Marengo D, Montag C, Sindermann C, Elhai JD, Settanni M (2021). Examining the links between active facebook use, received likes, self-esteem and happiness: a study using objective social media data. Telematics Informatics.

[72] Toma CL, Hancock JT (2013). Self-affirmation underlies facebook use. Pers Soc Psychol Bull.

[73] Lakey B, Orehek E (2011). Relational regulation theory: a new approach to explain the link between perceived social support and mental health. Psychol Rev.

[74] Brailovskaia J, Teismann T, Margraf J (2018). Cyberbullying, positive mental health and suicide ideation/behavior. Psychiatry Res.

[75] Calhoun CD, Stone KJ, Cobb AR, Patterson MW, Danielson CK, Bendezú JJ (2022). The role of social support in coping with psychological trauma: an integrated biopsychosocial model for posttraumatic stress recovery. Psychiatry Q.

[76] Tian Q (2016). Intergeneration social support affects the subjective well-being of the elderly: mediator roles of self-esteem and loneliness. J Health Psychol.

[77] Nick EA, Cole DA, Cho SJ, Smith DK, Carter TG, Zelkowitz RL (2018). The online social support scale: measure development and validation. Psychol Assess.

[78] Zhao C, Ding N, Yang X, Xu H, Lai X, Tu X, Lv Y, Xu D, Zhang G (2021). Longitudinal effects of stressful life events on problematic smartphone use and the mediating roles of mental health problems in chinese undergraduate students. Front Pub Health.

[79] Boyd dm, Ellison NB (2007). Social network sites: definition, history, and scholarship. J Computer-Mediated Commun.

[80] Wenninger H, Krasnova H, Buxmann P (2019). Understanding the role of social networking sites in the subjective well-being of users: a diary study. Eur J Inf Syst.

[81] Gilmour J, Machin T, Brownlow C, Jeffries C (2020). Facebook-based social support and health: a systematic review. Psychology of Popular Media.

[82] Zheng X, Wang Z, Chen H, Xie F (2021). The relationship between self-esteem and internet altruistic behavior: the mediating effect of online social support and its gender differences. Person Individual Diff.

[83] Porter AC, Zelkowitz RL, Gist DC, Cole DA (2019). Self-Evaluation and depressive symptoms: a latent variable analysis of self-esteem, shame-proneness, and self-criticism. J Psychopathol Behav Assess.

[84] Leary MR, Tambor ES, Terdal SK, Downs DL (1995). Self-esteem as an interpersonal monitor: the sociometer hypothesis. J Pers Soc Psychol.

[85] Wang Y, Nie R, Li Z, Zhou N (2018). WeChat Moments use and self-esteem among Chinese adults: the mediating roles of personal power and social acceptance and the moderating roles of gender and age. Personality Individ Differ.

[86] Karaca A, Yildirim N, Cangur S, Acikgoz F, Akkus D (2019). Relationship between mental health of nursing students and coping, self-esteem and social support. Nurse Educ Today.

[87] Jin, G. Lu, L. Zhang, X. Li. The mediating role of college students’ online social support in the relationship between self-esteem and online deviant behavior. Psychological Techniques and Application, 2017;5 (6), 327–333

[88] Rafferty R, Vander Ven T (2014). “I hate everything about you”: a qualitative examination of cyberbullying and on-line aggression in a college sample. Deviant Behav.

[89] Zhu C, Huang S, Evans R, Zhang W (2021). Cyberbullying among adolescents and children: a comprehensive review of the global situation, risk factors, and preventive measures. Front Pub Health.

[90] Hinduja S, Patchin JW (2017). Cultivating youth resilience to prevent bullying and cyberbullying victimization. Child Abuse Negl.

[91] Ladd GW, Ettekal I, Kochenderfer-Ladd B (2017). Peer victimization trajectories from kindergarten through high school: differential pathways for children’s school engagement and achievement?. J Educ Psychol.

[92] Akbulut Y, Erişti B (2011). Cyberbullying and victimisation among Turkish university students. Australas J Educ Technol.

[93] Hellfeldt K, López-Romero L, Andershed H (2019). Cyberbullying and psychological well-being in young adolescence: the potential protective mediation effects of social support from family, friends, and teachers. Int J Environ Res Public Health.

[94] Cénat JM, Blais M, Hébert M, Lavoie F, Guerrier M (2015). Correlates of bullying in Quebec high school students: the vulnerability of sexual-minority youth. J Affect Disord.

[95] Peled Y (2019). Cyberbullying and its influence on academic, social, and emotional development of undergraduate students. Heliyon.

[96] Maurya C, Muhammad T, Dhillon P, Maurya P (2022). The effects of cyberbullying victimization on depression and suicidal ideation among adolescents and young adults: a three year cohort study from India. BMC Psychiatry.

[97] Burns, M. L. Cyberbullying: reciprocal links with social anxiety, self-esteem and resilience in U.K. school children (Master's thesis, University of Chester, Chester, United Kingdom). 2017. Retrieved from https://chesterrep.openrepository.com/handle/10034/620963. Accessed 22 Aug 2023.

[98] Ding Z, Wang X, Liu Q (2018). The relationship between college students’ self-esteem and cyber aggressive behavior: the role of social anxiety and dual self-consciousness. Psychol Dev Educ.

[99] Pieschl S, Porsch T (2017). The complex relationship between cyberbullying and trust. Int J Dev Sustain.

[100] Denche-Zamorano Á, Barrios-Fernandez S, Galán-Arroyo C, Sánchez-González S, Montalva-Valenzuela F, Castillo-Paredes A, Rojo-Ramos J, Olivares PR (2022). Science mapping: a bibliometric analysis on cyberbullying and the psychological dimensions of the self. Int J Environ Res Public Health.

[101] Völlink T, Bolman CAW, Dehue F, Jacobs NCL (2013). Coping with cyberbullying: differences between victims, bully-victims and children not involved in bullying. J Commun App Soc Psychol.

[102] Brislin RW (1976). Comparative research methodology: cross-cultural studies. Int J Psychol.

[103] Rosen LD, Whaling K, Carrier LM, Cheever NA, Rokkum J (2013). The media and technology usage and attitudes scale: an empirical investigation. Comput Hum Behav.

[104] Barton BA, Adams KS, Browne BL, Arrastia-Chisholm MC (2021). The effects of social media usage on attention, motivation, and academic performance. Act Learn High Educ.

[105] Ybarra ML, Espelage DL, Mitchell KJ (2007). The co-occurrence of Internet harassment and unwanted sexual solicitation victimization and perpetration: associations with psychosocial indicators. The J Adolescent Health.

[106] Jiang H, Chen G, Wang T (2017). Relationship between belief in a just world and Internet altruistic behavior in a sample of Chinese undergraduates: Multiple mediating roles of gratitude and self-esteem. Personality Individ Differ.

[107] Zhou Z, Cheng Q (2022). Measuring online social support: development and validation of a short form for Chinese adolescents. Int J Environ Res Public Health.

[108] Li R-H (2014). Reliability and validity of a shorter Chinese version for Ryff’s psychological well-being scale. Health Educ J.

[109] Tan Y, Huang C, Geng Y, Cheung SP, Zhang S (2021). Psychological well-being in Chinese college students during the COVID-19 pandemic: roles of resilience and environmental stress. Front Psychol.

[110] Zhang Y, Carciofo R (2021). Assessing the wellbeing of Chinese university students: validation of a Chinese version of the college student subjective wellbeing questionnaire. BMC psychology.

[111] Hayes AF. Introduction to Mediation, Moderation, and Conditional Process Analysis. A Regression-Based Approach (2nd ed.). New York: The Guilford Press; 2018.

[112] Aiken, L. S., & West, S. G. Multiple regression: Testing and interpreting interactions. Sage Publications, Inc. 1991

[113] Hair J, Hollingsworth CL, Randolph AB, Chong AYL (2017). An updated and expanded assessment of PLS-SEM in information systems research. Ind Manag Data Syst.

[114] Fornell C, Larcker DF (1981). Evaluating structural equation models with unobservable variables and measurement error. J Mark Res.

[115] Hair, J. F, Hult, G. Tomas M, Ringle, C. M, & Sarstedt, M. A primer on partial least squares structural equation modeling (PLS-SEM). 2016;2nd ed. Los Angeles: SAGE.

[116] Podsakoff PM, MacKenzie SB, Podsakoff NP (2012). Sources of method bias in social science research and recommendations on how to control it. Annu Rev Psychol.

[117] Wellman B (2001). Computer networks as social networks. Science.

[118] Diener E, Diener M (1995). Cross-cultural correlates of life satisfaction and self-esteem. J Pers Soc Psychol.

[119] Steger MF, Frazier P, Oishi S, Kaler M (2006). The meaning in life questionnaire: assessing the presence of and search for meaning in life. J Couns Psychol.

[120] Steele CM (1988). The psychology of self-affirmation: Sustaining the integrity of the self. In L. Berkowitz (Ed.). Soc Psychol Stud Self.

[121] Leary, M. R., & Baumeister, R. F. The nature and function of self-esteem: Sociometer theory. In M. P. Zanna (Ed.), Advances in experimental social psychology, 2000; Vol. 32, pp. 1–62. Academic Press. 10.1016/S0065-2601(00)80003-9

[122] Zheng Q, Yao T, Fan X (2016). Improving customer well-being through two-way online social support. J Serv Theory Pract.

[123] Nabi RL, Prestin A, So J (2013). Facebook friends with (health) benefits? Exploring social network site use and perceptions of social support, stress, and well-being. Cyberpsychol Behav Soc Netw.

[124] Indian M, Grieve R (2014). When facebook is easier than face-to-face: Social support derived from facebook in socially anxious individuals. Personality Individ Differ.

[125] Neira CJB, Barber BL (2014). Social networking site use: Linked to adolescents' social self-concept, self-esteem, and depressed mood. Aust J Psychol.

[126] Woods HC, Scott H (2016). #Sleepyteens: Social media use in adolescence is associated with poor sleep quality, anxiety, depression and low self-esteem. J Adolesc.

[127] Harter, S. The construction of the self: Developmental and sociocultural foundations (2nd ed.). The Guilford Press. 2012

[128] Zhang H, Guan L, Qi M, Yang J (2013). Self-esteem modulates the time course of self-positivity bias in explicit self-evaluation. PLoS ONE.

[129] Naslund JA, Aschbrenner KA, Marsch LA, Bartels SJ (2016). The future of mental health care: peer-to-peer support and social media. Epidemiol Psychiatric Sci.

[130] Reining, Lauren; Drouin, Michelle; Toscos, Tammy; and Mirro, Michael J. "College students in distress: Can social media be a source of social support?". Presentations and Events. 2018;7. https://researchrepository.parkviewhealth.org/presentations/7

[131] Cao X, Khan AN, Zaigham GHK, Khan NA (2019). The stimulators of social media fatigue among students: role of moral disengagement. J Educ Computing Res.

[132] Sampasa-Kanyinga H, Hamilton HA (2015). Social networking sites and mental health problems in adolescents: The mediating role of cyberbullying victimization. European Psychiatry.

[133] Valkenburg PM, Peter J (2013). The differential susceptibility to media effects model. J Commun.

[134] Gerbner G, Gross L (1976). Living with television: the violence profile. J Commun.

[135] Heiman T, Olenik-Shemesh D, Frank G (2019). Patterns of coping with cyberbullying: emotional, behavioral, and strategic coping reactions among middle school students. Violence Vict.

[136] Valkenburg PM (2022). Social media use and well-being: what we know and what we need to know. Curr Opinion Psychol.

[137] Yang CC, Holden SM, Ariati J (2021). Social media and psychological well-being among youth: the multidimensional model of social media use. Clin Child Fam Psychol Rev.

[138] Zhou X, Snoswell CL, Harding LE, Bambling M, Edirippulige S, Bai X, Smith AC (2020). The role of telehealth in reducing the mental health burden from COVID-19. Telemed E-Health.

